# 2 What You Wear Underneath Your Gear Matters

**DOI:** 10.1093/jbcr/iraf019.002

**Published:** 2025-04-01

**Authors:** Michael Feldman, E Gerald, Joshua Petteway, Denise Statham, Prabhu Senthil-Kumar

**Affiliations:** Virginia Commonwealth University; Chesterfield County Fire and EMS, Chesterfield, Virginia; DuPont de Nemours, Inc; Workwear Outfitters; Evans-Haynes Burn Center

## Abstract

**Introduction:**

Fire fighter gear is required to be no melt/drip consistent with the NFPA 1975 Station Wear Standard in order to reduce the risk of injury. This standard does not address what is worn underneath the gear. Anecdotal evidence suggests that polyester or blends tend to melt. Traditional testing has been performed on upright thermal manikins fitted with sensors that determine burn injury and garment performance. To date, this has not been reported in tests that replicate an actual burn scenario.

The purpose of this study is to create a model that more accurately recreates a flashover event in order to determine the effect of what is worn underneath a Fire Fighter’s gear and how it relates to injury potential.

**Methods:**

We designed three scenarios utilizing “sand” mannikins outfitted with commercially available turnout gear. Each mannikin was outfitted with 9 “puck” style sensors and 2 backups. The sensors were placed in the same locations for each manikin: center chest, center lower back, and outside under the air pack. The sensors were then modified with extended T-type thermocouple wire, fitted with fireproof insulative sheathing and tape, and the wire ends mated with ceramic plug ends to connect to a mobile data acquisition box. The mannikins were then placed into Flashover containers with infrared cameras to record the event. Data was collected at 30, 20, and 1-2 second flashover events.

**Results:**

Our results show that the polyester and poly/cotton blends melt under commercially available turnout gear during flashover events. There is a direct correlation between the presence of melted polyester and blend materials and worsened injury to the mannikin simulating severe burn injuries when compared to cotton undergarments.

**Conclusions:**

Polyester and poly/cotton blends melt under turnout gear during flashover events leading to increased injury to our mannikin model. This data supports the avoidance of these materials under Firefighter gear as a way to reduce risk of significant burn injury. We are working to raise awareness of that what you wear underneath your gear matters as our Firefighters matter to us.

**Applicability of Research to Practice:**

Synthetic base layers melt during firefighting conditions. This information should be used to help inform and protect our Firefighter community.

**Funding for the Study:**

The turnout gear, Manikins, sensors, and undergarments were all donated for this study.

